# Fostering Equity in Dementia Care: The Vital Role of Culturally Sensitive Technology

**DOI:** 10.1002/alz.086704

**Published:** 2025-01-09

**Authors:** Arshia A Khan, Rana Z Imtiaz

**Affiliations:** ^1^ University of Minnesota Duluth, Duluth, MN USA; ^2^ Kentucky College of Osteopathic Medicine, PikeVille, KY USA

## Abstract

This perspective abstract highlights the critical demand for culturally sensitive technology in dementia caregiving due to the heightened risk and unique challenges faced by individuals from diverse backgrounds. The escalating prevalence of dementia necessitates a caregiving paradigm shift, especially in the context of diverse cultural backgrounds. Research consistently underscores the existence of cultural disparities in dementia risk, diagnosis, and care outcomes, emphasizing the urgent need for culturally sensitive technology in dementia care.

Individuals from diverse cultural backgrounds often face unique challenges in accessing and receiving adequate dementia care. These challenges may stem from variations in communication styles, beliefs, and societal attitudes toward dementia. Culturally sensitive technology has the potential to bridge these gaps by tailoring interventions to align with specific cultural contexts. It can enhance communication, understanding, and overall caregiving effectiveness, providing personalized support that respects the cultural nuances of those affected by dementia.

Moreover, incorporating cultural sensitivity into technology design not only addresses disparities but also contributes to more inclusive healthcare practices. Culturally tailored technologies can empower caregivers with tools that recognize and respect diverse perspectives, fostering a more collaborative and patient‐centered approach. In essence, the need for culturally sensitive technology in dementia care is paramount to ensuring equitable access, improving outcomes, and promoting a holistic understanding of the multifaceted challenges faced by individuals from diverse cultural backgrounds affected by dementia. This imperative aligns with the broader goal of enhancing the quality of life for all individuals affected by dementia while fostering a more inclusive and compassionate healthcare environment.

